# Missed Wrist Septic Arthritis: A Cautionary Case Report in a Postpartum Patient With Complement Deficiency

**DOI:** 10.7759/cureus.109002

**Published:** 2026-05-17

**Authors:** Isabella Bozzo, Dominique Tremblay, Edward J Harvey, Janius Tsang

**Affiliations:** 1 Orthopedic Surgery, McGill University, Montreal, CAN; 2 Plastic and Reconstructive Surgery, Université de Montréal, Montréal, CAN; 3 Orthopaedic Surgery, McGill University, Montreal, CAN; 4 Anesthesiology, McGill University, Montreal, CAN

**Keywords:** carpus arthropathy, complement deficiency, postpartum infection, preseptal orbital cellulitis, wrist septic arthritis

## Abstract

A 38-year-old female, only known for beta-thalassemia minor trait, presented 3.5 weeks postpartum with six days of worsening right wrist pain and sepsis. Initial misdiagnosis resulted in delayed surgical debridement of her wrist after two months. Additionally, a concurrent orbital pre-septal cellulitis with different bacteria aided in the diagnosis of an underlying postpartum complement deficiency predisposing her to multiple infections with different organisms in the acute postpartum period. Follow-up at one year and eight years demonstrated significant functional limitations, reduced range of motion, and radiographic evidence of pan-carpal destructive arthropathy with fusion of the carpus. Septic arthritis is a surgical emergency; however, postpartum immunodeficiency is an unreported condition that must be recognized due to the risk of developing multisystem infections and may lead to delayed treatment with devastating sequelae.

## Introduction

Septic arthritis, specifically of the wrist, is a relatively uncommon pathology that carries a significant morbidity to patients if missed [1-5]. The overall incidence of wrist septic arthritis is 5%, representing 23% of upper extremity infections, and a single institution demonstrated only 40 cases over an 11-year period [2,3]. Most patients present with atraumatic wrist pain, decreased range of motion (ROM), erythema, and warmth, and some may appear systemically unwell (febrile, septic shock) [2,6]. The mainstay of treatment relies on identification of the organism, followed by targeted antibiotic therapy and surgical drainage [1-3,7]. A missed diagnosis may lead to carpal cartilage and joint destruction within eight hours, as well as severe life-threatening bacteremia with sepsis [1,3,8,9].

Complement deficiency refers to the absence or dysfunction of one or many proteins in the complement cascade, which my predispose patients to recurrent or severe bacterial infections. Postpartum infections affecting multiple systems are uncommon complications attributed to the relative deficiency of complement levels as well as complement reconstitution syndrome, whereby the reactivation of the immune system triggers a dysregulated response, leading to a paradoxical clinical worsening [10-15].

While there are a few case reports of postpartum septic arthritis, affecting the knee, shoulder, hip, and pubic symphysis, none of these patients was found to have immune deficiencies [16-20]. To the authors’ knowledge, there are no published cases of postpartum C3 complement deficiency presenting as septic arthritis, more specifically in the wrist. This case report aims to describe a unique manifestation of an initially missed diagnosis of septic arthritis of the wrist with *Streptococcus pyogenes*, with an eight-year follow-up, and recommendations for appropriate multidisciplinary management.

## Case presentation

A 38-year-old female, known for asymptomatic anemia due to beta-thalassemia minor trait, presented to a Canadian tertiary care hospital emergency department three and a half weeks postpartum following an uncomplicated vaginal delivery with a six-day history of worsening right wrist pain. She took no medications, had no history of substance misuse, and had no allergies. There was also no recent history of trauma.

**Figure 1:**
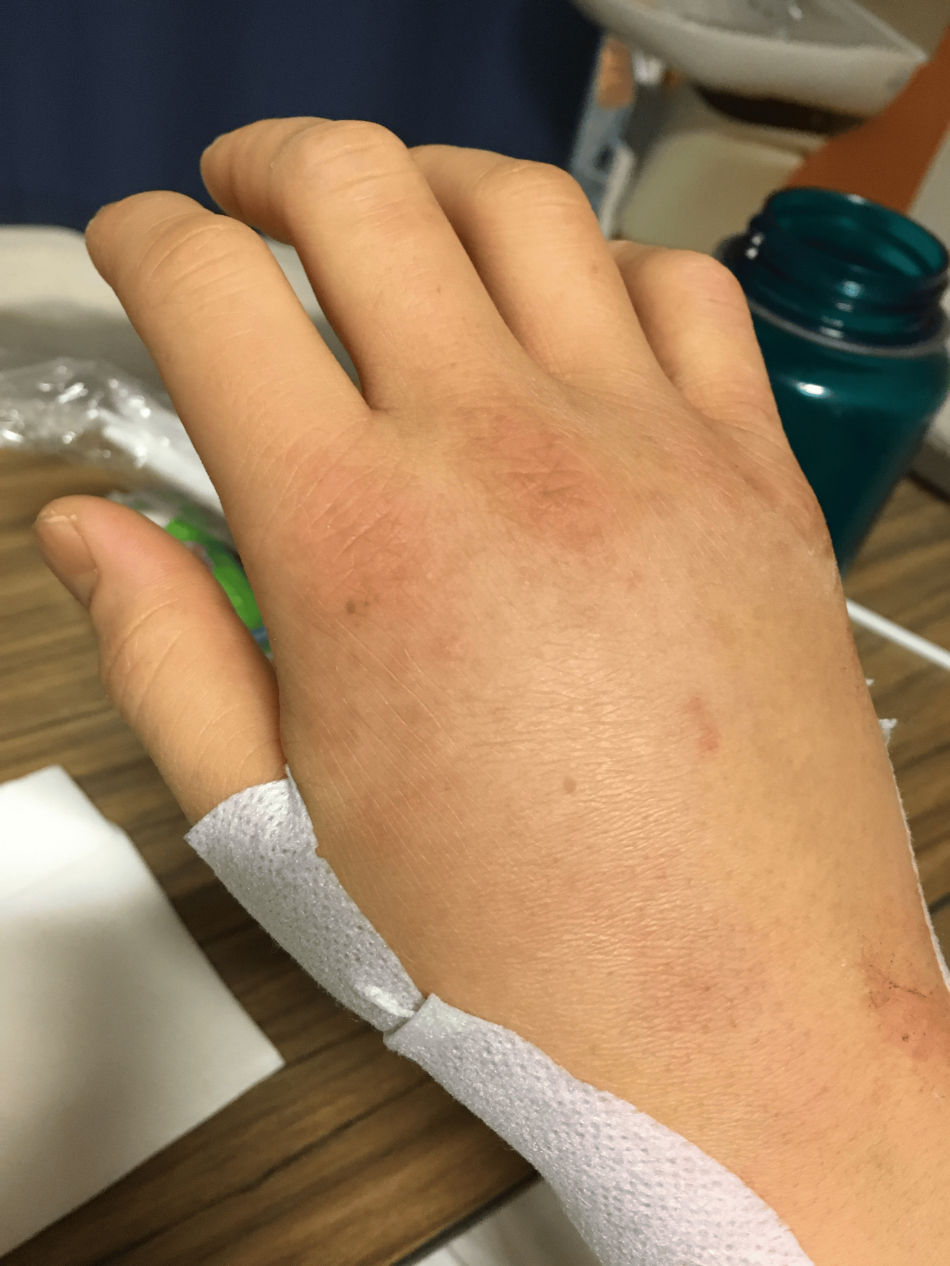
Right wrist eight hours after initial presentation to the emergency department.

Her wrist pain onset six days prior to presentation was accompanied by rigors, generalized malaise, anorexia, and vomiting. Fifteen hours following the onset of her systemic symptoms, she reported severe aching pain in her right wrist waking her from sleep. On initial examination, passive and active ROM were limited by pain, paresthesia was present in the median nerve, and Tinel and Phalen’s signs were positive; however, there was no erythema or swelling of the wrist (Figure 1). 

The provisional diagnosis was acute carpal tunnel syndrome, and she underwent an acute decompression under local anesthesia within 24 hours of presentation by a board-certified plastic surgeon. She was discharged following the decompression.

Four days after the carpal tunnel release, she presented again to the same emergency room with new diffuse abdominal pain and distention, oliguria, and 6 kg of weight gain. Initial vital signs were consistent with shock (heart rate of 115, blood pressure of 107/60, respiratory rate of 22 breaths/min, oxygen saturation of 97% in ambient air, and rectal temperature of 36.9 degrees Celsius). On physical examination, the right wrist demonstrated a diffusely edematous dorsum with pain to palpation of the metacarpophalangeal joints and dorsal hand, and active and passive finger ROM was limited by pain. The four Kanavel signs were positive: 1) fusiform swelling of the digits, 2) flexed posture of the digit, 3) pain with passive extension, and 4) tenderness along the volar flexor tendon sheath. The surgical carpal tunnel incisions were clean and intact. Re-examination after 18 hours demonstrated increased edema and blue-brown discoloration without frank necrosis. Additionally, she was noted to have mild icterus. The abdominal examination revealed diffuse tenderness with voluntary guarding in both lower quadrants, and the gynecological examination provoked only mild discomfort.

**Figure 2:**
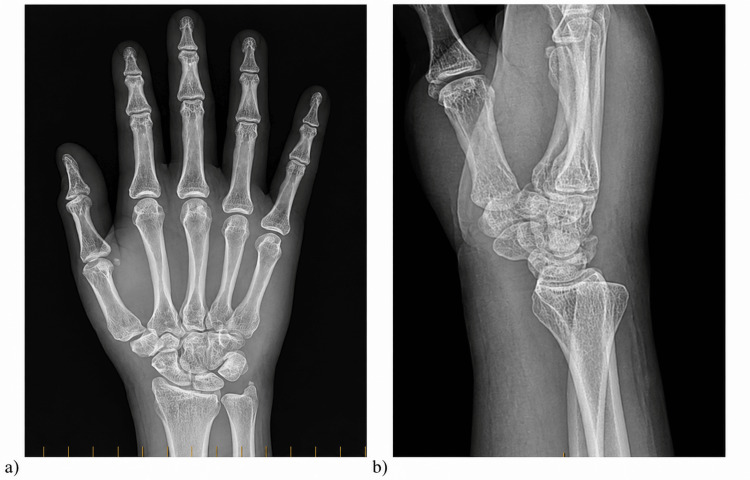
Plain radiographs obtained 10 days after the initial onset of symptoms without evidence of chondrolysis or joint destruction: a) anteroposterior view and b) lateral view.

Laboratory investigations showed marked leukocytosis with neutrophilia (white blood cell count of 30.0 x 10^9^, 80% neutrophils, normal range: 4.0-10.0 x 10^9^) and elevated C-reactive protein (365 mg/L, normal range < 5 mg/L). Blood cultures grew gram-positive cocci in chains after 15 hours and ultimately confirmed *Streptococcus pyogenes*. The key diagnostic turning point was that septic arthritis was not suspected clinically, and a joint aspirate was not obtained at this time. Plain radiographs were reported as normal without evidence of joint destruction or chondrolysis (Figures 2a, 2b).

Computed tomography (CT) and magnetic resonance imaging were deemed unnecessary based on the working diagnosis and concerns for confounding from post-surgical changes.

At 18 hours, she was diagnosed with necrotizing fasciitis and underwent extensive hand fasciotomies and surgical debridement. Intraoperatively, the fascia, muscles, and nerves were healthy, without evidence of necrosis. Seven tissue and exudate specimens were sent for culture. However, the wrist joint capsule was not opened for drainage under the assumption that this was an isolated skin and soft tissue infection, not septic arthritis, and the treating surgeon wanted to avoid seeding the joint. The intraoperative diagnosis was early necrotizing soft tissue infection. The fasciotomies were left open, and the patient was transferred to the intensive care unit (ICU) for observation.

She received empiric broad-spectrum antibiotic therapy with piperacillin-tazobactam, vancomycin, and clindamycin for 48 hours’ duration, which was subsequently narrowed to penicillin and then ceftriaxone following *Streptococcus pyogenes* sensitivities. To rule out alternative foci of infection, a thoracoabdominal CT scan and transthoracic echocardiography were performed, both of which were within normal limits. The patient stayed in the ICU for 18 hours and was discharged home after three additional days in the hospital. A 14-day course of intravenous ceftriaxone was completed as an outpatient.

Despite the improvement in her general state of health, the patient continued to have significant right wrist pain. The pain was poorly controlled with narcotic analgesia. Fourteen days after the second extensive surgery, the open wounds were closed primarily under axillary block. It was noted that passive wrist ROM was significantly reduced even under regional anesthesia. Given this mechanical limitation of movement and persistent pain, it was presumed that a post-infection complex regional pain syndrome (CRPS) was developing. The patient was encouraged to undergo intensive physiotherapy to avoid the onset of CRPS.

**Figure 3:**
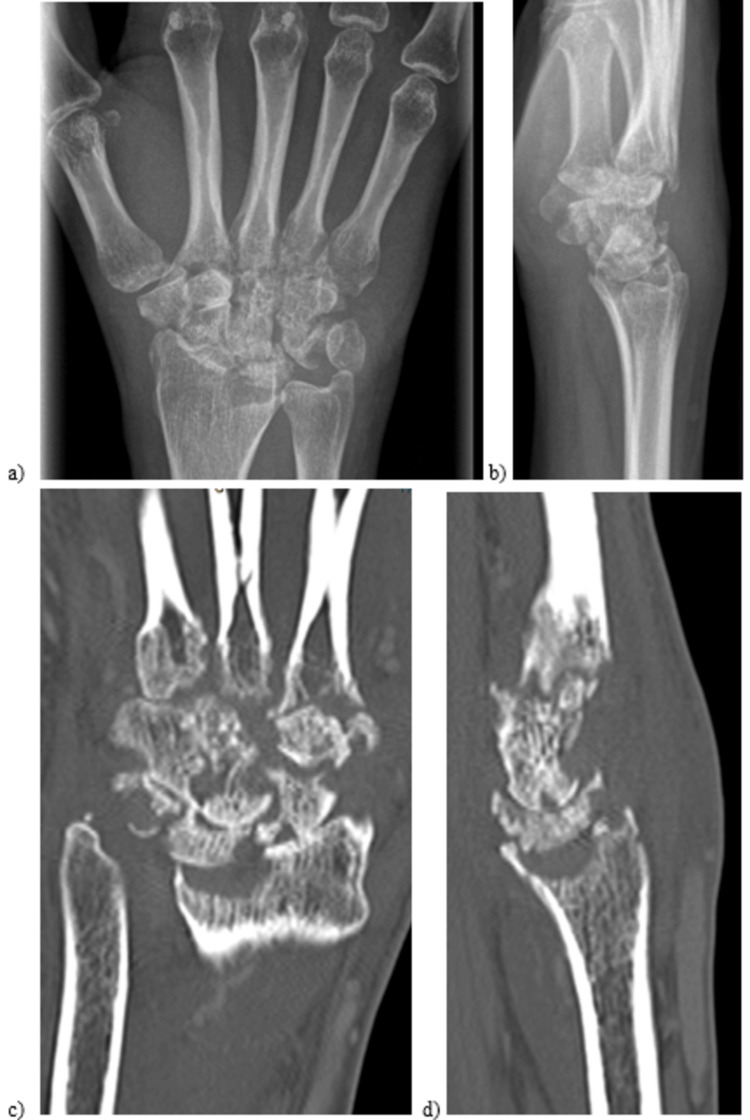
Repeat imaging obtained two months following the initial onset of symptoms demonstrating erosive damage and diffuse destruction of the carpus, radiocarpal joint, and carpometacarpal joints, with carpal subluxation into the eroded radius. Right wrist radiograph: a) anteroposterior view and b) lateral view. Computed tomography: c) coronal view and d) sagittal view.

Two months after the wound closure, the right wrist was noted to have residual swelling, marked limitations in active and passive ROM, and persistent severe pain on palpation. The patient was otherwise well. The Disabilities of the Arm, Shoulder, and Hand (DASH) score was 86.7. A plain wrist radiograph and CT at this time showed severe, diffuse joint destruction, chondrolysis, and osteoarthritis affecting the radiocarpal, midcarpal, and carpometacarpal joints (Figures 3a-3d).

The radiographic changes were consistent with the sequelae of septic arthritis and associated osteomyelitis. Fluoroscopic-guided joint aspiration was performed with minimal fluid return, and the final cultures were negative for a causative organism.

**Figure 4:**
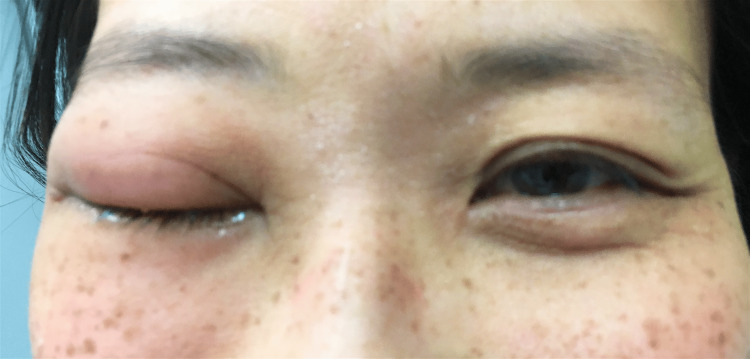
Right eye preseptal cellulitis due to Streptococcus pneumoniae that developed two months after initially presenting with wrist septic arthritis.

A decision was made to proceed with an irrigation and debridement of the right wrist; however, while waiting for the lavage, she developed atraumatic pain and swelling in her right eyelid after an episode of allergic rhinitis and conjunctivitis (Figure 4).

She was diagnosed with right preseptal cellulitis, was treated with piperacillin-tazobactam as empiric therapy, and went for simultaneous lavage and debridement of the right wrist and right eyelid. For the wrist, no bacteria were cultured nor bacterial deoxyribonucleic acid detected with polymerase chain reaction analysis of the wrist tissue cultures. *Streptococcus pneumoniae* was obtained from cultures of the right eyelid samples. Since both strains were sensitive to penicillin, the patient received six weeks of intravenous penicillin.

Immunology was subsequently consulted given the simultaneous infections with two different bacterial strains. Their input aided in the diagnosis of C3 complement deficiency, predisposing the patient to recurrent infections in the postpartum period. No additional treatment was required. No further infections were reported over the following year, and extensive physiotherapy and occupational therapy were undertaken to manage the wrist disability.

**Figure 5:**
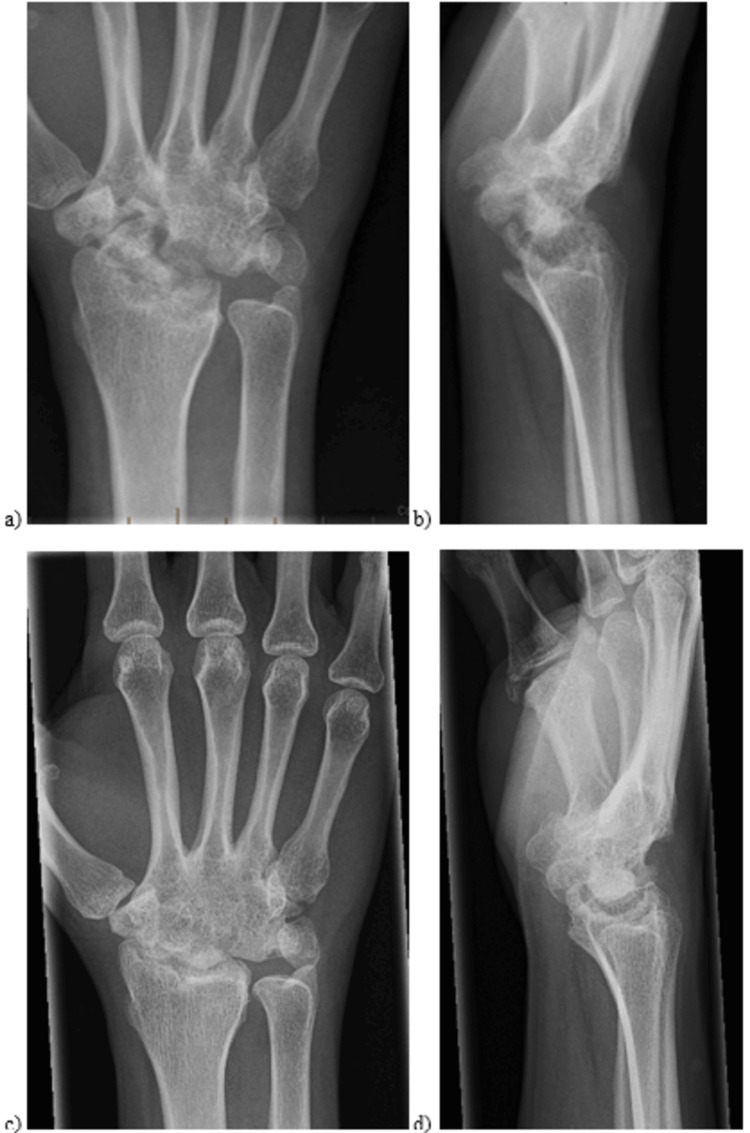
Radiographic follow-up at one year and eight years showing osseous remodeling with osseous fusion at the intercarpal bones and carpometacarpal joint with degenerative changes of the radiocarpal joint. Radiograph at the one-year follow-up: a) anteroposterior view and b) lateral view. Radiograph at the eight-year follow-up: c) anteroposterior view and d) lateral view.

At the one-year follow-up, there was significant functional limitations compared to the contralateral side. Wrist passive and active flexion was limited to neutral, maximum passive extension was 20°, and there was no active or passive ulnar or radial deviation. Pronation was fully preserved, but supination was limited to 45°. The DASH score was 40.8. Radiographs showed pan carpal destruction and fusion proximal carpals to the radial and ulnar heads and fusion of the distal carpals to the metacarpal heads (Figures 5a-5d).

At this point, she resumed a gradual return to work plan as an anesthesiologist, and she continued to undergo treatments with physiotherapy.

**Figure 6:**
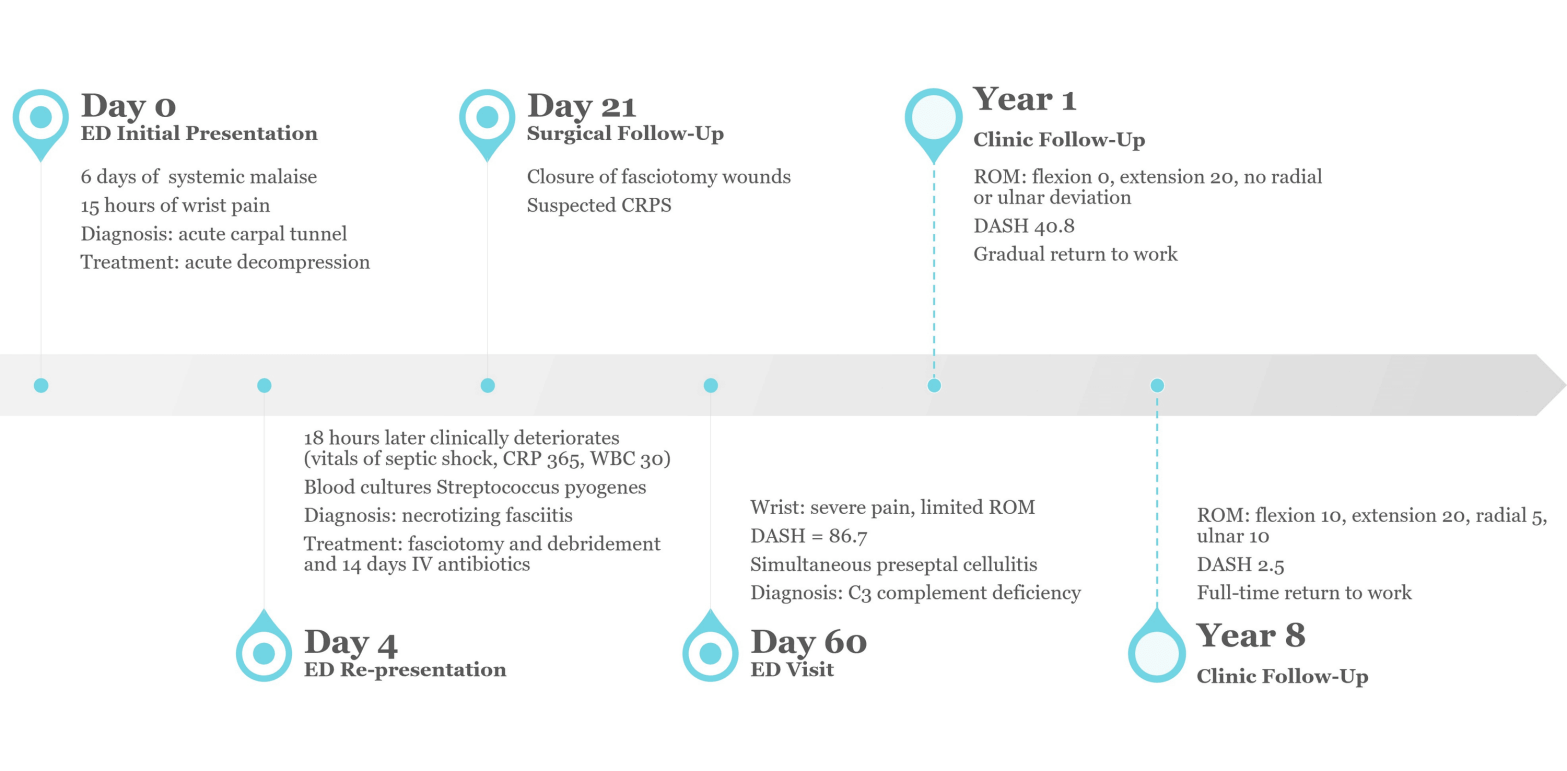
Timeline of key events with pertinent laboratory and imaging findings, as well as medical and surgical treatments. Image credits: The image was created by the authors using Microsoft PowerPoint (Microsoft Corporation, Redmond, WA) CRP, C-reactive protein; CRPS, complex regional pain syndrome; DASH, Disabilities of the Arm, Shoulder, and Hand; ROM, range of motion; WBC, white blood cell

At the eight-year follow up, clinical examination showed ongoing limitations in ROM (maximum passive and active wrist flexion 10°, extension 30°, radial deviation 5° and ulnar deviation 10°, and forearm pronation 90° and supination 60°). Radiographs demonstrate chronic osseous remodeling with osseous fusion at the intercarpal bones and carpometacarpal joint, with degenerative changes of the radiocarpal joint (Figures 5A-5D). The patient’s pain had improved, the DASH score improved to 2.5, and she returned to work full-time as an anesthesiologist. A timeline summarizing the key events of this case, with pertinent laboratory, imaging, and treatments, is presented in Figure 6.

## Discussion

A missed diagnosis of wrist septic arthritis may be devastating for patients [1-3]. As shown in this case, joint infections may lead to chondrolysis, joint destruction, and ultimately fusion and/or pseudoarthrosis if not treated promptly with surgical drainage and lavage. Chondrolysis was shown to begin as early as eight hours post-infection, and the best patient functional outcomes have been demonstrated when arthrotomy and lavage are performed within the first 10 hours of infection onset [2]. With delayed treatment, this may lead to long-term functional limitations with disability and inability to return to work [9]. Unfortunately, according to the literature, it is not uncommon to have misdiagnoses and prolonged delays to surgery for wrist septic arthritis, with a mean of 6.8 days from presentation to debridement following a 11-year review in a retrospective review of a single institution [3].

Long-term sequelae of wrist septic are not well known; however, a retrospective cohort study of 18 patients with a median follow-up of 44 months (with three immunocompromised patients), who underwent surgical debridement, demonstrated decreased ROM by 49% and decreased grip strength by 70% compared with the contralateral side and a DASH score of 34 [1]. As with our patient, reduced ROM compared to the contralateral wrist was identified; however, the DASH score did improve from the one- to eight-year follow-up. While our patient did eventually return to work, this was not without an initial one-year period of disability and gradual return to work physiotherapy plan.

High rates of confounding differential diagnoses, such as cellulitis or soft tissue infections, spontaneous hematomas, rheumatoid or other inflammatory arthritis, and gout or pseudogout (crystal arthropathy), were attributed to the delay in diagnosis and treatment, as was evidenced by this case with an initial working diagnosis of necrotizing skin and soft tissue infection [2,6]. The most common risk factor for septic arthritis reported was immunocompromise, with a prevalence as high as 80% in patients with wrist septic arthritis; some examples include blood dyscrasias, malignancy, end-stage renal disease, and immunosuppressive medications for chronic systemic disease [3,6]. Furthermore, failure to isolate a bacterial etiology in wrist tissue may suggest a possible autoimmune mechanism via molecular mimicry. While the C3 complement deficiency was presumed to be the underlying risk factor predisposing this patient to wrist septic arthritis and preseptal cellulitis, the authors acknowledge the limitation that causality between postpartum immune changes and septic arthritis cannot be definitively established from a single case report.

## Conclusions

This case report reviews an eight-year follow-up of a 38-year-old female with delayed diagnosis and treatment of wrist septic arthritis resulting from a new immunodeficiency. The sequelae of missed septic arthritis can be devastating for patients, including loss of movement, chronic pain, and disability. Postpartum complement deficiencies are not well documented in the literature as a risk factor for septic arthritis and have never been reported for wrist septic arthritis. It has been reported in obstetric literature that patients without risk factors for concordant infections presenting with recurrent postpartum infections, such as respiratory infections, should be screened for complement deficiencies. Therefore, the primary consultant in the management of septic arthritis should consider this manifestation in postpartum patients and consider the possibility of an underlying immunodeficiency, as well as promptly consulting specialist colleagues in immunology.
